# Risk stratification tools for patients with syncope in emergency medical services and emergency departments: a scoping review

**DOI:** 10.1186/s13049-023-01102-z

**Published:** 2023-09-18

**Authors:** Lucia G. uit het Broek, B. Bastiaan A. Ort, Hester Vermeulen, Thomas Pelgrim, Lilian C.M. Vloet, Sivera A.A. Berben

**Affiliations:** 1 Research Department of Emergency and Critical Care, School of Health Studies, HAN University of Applied Sciences, Nijmegen, The Netherlands; 2 Scientific Institute for Quality of Healthcare, Radboud Institute for Health Sciences, Radboud university medical center, Nijmegen, The Netherlands; 3 Department of Primary and Community Care, Radboud Institute for Health Sciences, Radboud university medical center, Nijmegen, The Netherlands

**Keywords:** Syncope, Risk stratification, Clinical decision rule, Emergency medical services, Emergency department, Decision-making

## Abstract

**Background:**

Patients with a syncope constitute a challenge for risk stratification in (prehospital) emergency care. Professionals in EMS and ED need to differentiate the high-risk from the low-risk syncope patient, with limited time and resources. Clinical decision rules (CDRs) are designed to support professionals in risk stratification and clinical decision-making. Current CDRs seem unable to meet the standards to be used in the chain of emergency care. However, the need for a structured approach for syncope patients remains. We aimed to generate a broad overview of the available risk stratification tools and identify key elements, scoring systems and measurement properties of these tools.

**Methods:**

We performed a scoping review with a literature search in MEDLINE, CINAHL, Pubmed, Embase, Cochrane and Web of Science from January 2010 to May 2022. Study selection was done by two researchers independently and was supervised by a third researcher. Data extraction was performed through a data extraction form, and data were summarised through descriptive synthesis. A quality assessment of included studies was performed using a generic quality assessment tool for quantitative research and the AMSTAR-2 for systematic reviews.

**Results:**

The literature search identified 5385 unique studies; 38 were included in the review. We discovered 19 risk stratification tools, one of which was established in EMS patient care. One-third of risk stratification tools have been validated. Two main approaches for the application of the tools were identified. Elements of the tools were categorised in history taking, physical examination, electrocardiogram, additional examinations and other variables. Evaluation of measurement properties showed that negative and positive predictive value was used in half of the studies to assess the accuracy of tools.

**Conclusion:**

A total of 19 risk stratification tools for syncope patients were identified. They were primarily established in ED patient care; most are not validated properly. Key elements in the risk stratification related to a potential cardiac problem as cause for the syncope. These insights provide directions for the key elements of a risk stratification tool and for a more advanced process to validate risk stratification tools.

**Supplementary Information:**

The online version contains supplementary material available at 10.1186/s13049-023-01102-z.

## Introduction

Transient loss of consciousness (T-LOC) is one of the most common symptoms of patients seeking prehospital emergency medical care and constitutes a major challenge for risk stratification in (prehospital) emergency care. Patients with a T-LOC account for up to 10% of emergency medical services (EMS) emergency calls, within non-conveyance rates up to 16.7%, and makeup to 3% of all emergency department (ED) visits [1–6]. The two main groups of T-LOC are T-LOC due to head trauma and ‘non-traumatic’ T-LOC. Non-traumatic T-LOC is further divided into syncope, epileptic seizures, psychogenic T-LOC, and a group of rare causes, of which syncope is the most common [7, 8]. Syncope is defined as a T-LOC due to cerebral hypoperfusion and is characterised by a rapid onset, short duration, and complete spontaneous recovery [7]. The aetiology of syncope varies from the relatively harmless vasovagal syncope to potentially fatal heart disease [8].

Professionals in the EMS and the ED (chain of emergency care) face the difficulty of identifying signs and symptoms of potential underlying etiology and need to differentiate between the high-risk syncope that will develop serious short-term outcomes from the large majority of low-risk syncope [9]. This risk stratification is complicated because the patient often has no residual complaints of the T-LOC when examined by an EMS or ED professional. In addition, professionals in EMS do not have the time or resources to perform various clinical tests and monitor the patient for an extended time before making a clinical decision [10].

Clinical decision rules (CDRs) are designed to support professionals in risk stratification and clinical decision-making [11]. Regarding syncope patients, CDRs have been proposed to support professionals in the chain of emergency care in clinical decision-making [5, 12]. A CDR can help identify low-risk syncope patients in the EMS setting who can benefit from referral to an outpatient clinic or general practitioner instead of transfer to an ED [12]. Likewise, it can help professionals in the ED to identify syncope patients who can be discharged home safely [5, 13]. Using a CDR could reduce the workload in the chain of emergency care, thereby reducing cost and improving the utilisation of increasingly precious emergency resources [12]. An accurate CDR could contribute to appropriate and safe care usage and providing the proper care at the right time.

Multiple CDRs for risk stratification and decision-making in syncope patients have been developed in the last two decades. However, systematic reviews show that the CDRs have not been validated or are poorly validated and are not generalisable. In general, the CDRs do not perform better than clinical judgement [14–16]. In addition, the systematic reviews indicate a large heterogeneity observed between the studies, limiting the possibilities of quantitative comparison [15, 16]. Moreover, these reviews have not revealed CDRs for syncope patients in EMS patient care. A review covering CDRs usable in the EMS is lacking to our knowledge.

Current CDRs seem unable to meet the standards to be used in the chain of emergency care. However, the need for a valid CDR or ways for a structured approach for syncope patients remains. Insight into current elements of risk stratification tools can contribute to developing a valid CDR. To address this, we performed a scoping review to generate a broad overview of available risk stratification tools and included elements in EMS and ED patient care. These elements can be used in the development of future CDRs. Therefore, the aim of this scoping review was to:

1. Identify risk stratification tools for syncope patients in EMS and ED,
2. Identify key elements, scoring systems, and measurement properties of these risk stratification tools for syncope patients in the chain of emergency care.

## Method

### Protocol and registration

The scoping review was conducted following the methodological framework of Arksey and O’Malley [17] and the Joanna Briggs Institute [18]. A scoping review protocol was developed with a medical librarian (TP). The Preferred Reporting Items for Systematic Reviews and Meta-analysis Extension for Scoping Reviews (PRISMA-ScR) were used for reporting [19].

### Search strategy

First, an initial search was conducted in MEDLINE and the Cumulative Index to Nursing and Allied Health Literature (CINAHL) to identify relevant keywords and index terms. Based on the initial search, the finalised search strategy was built with the medical librarian’s (TP) help. The following terms were used (including synonyms and closely related words) as keywords, index terms, or free-text words to represent the concepts: syncope, triage or tool, and EMS or ED. Six databases were searched: MEDLINE (EBSCO), CINAHL (EBSCO), Pubmed, Embase (OVID), Cochrane Central, and Web of Science Core Collection. We limited our search from January 2010 to the 12th of May 2022 because non-conveyance decision-making in EMS has been a tendency of the last decade and, therefore, the possible need for a risk stratification tool [20]. In addition, Serrano et al. [14] conducted their literature search until November 2009. The results were uploaded into EndNote to duplicate removal. The de-duplication of the database search results was conducted following the method of Bramer et al. [21]. Additionally, the researchers searched the reference lists of included studies. The original derivation study was manually searched if an included article described a tool created before 2010. Grey literature was not included in the search strategy. The search strategy is presented in additional file 1.

### Study selection

The title and abstracts of the studies were independently screened by two researchers (LB, BO) using Rayyan (https://rayyan.ai/cite). The researchers calibrated their screening process after 20, 100, 500, and 2500 screened titles and abstracts. Subsequently, the two researchers independently assessed the full text of identified articles. The researchers calibrated their screening process after ten screened articles. The process was supervised by a third researcher (SB), who acted as a third reviewer in case of disagreement between the two researchers until a consensus was reached.

Inclusion criteria were (i) the population consisted of patients with syncope or T-LOC; (ii) the context consisted of EMS or ED patient care, and (iii) the studies described a tool to support the risk stratification of syncope patients of serious short-term outcomes (maximum 30 days) or cardiac syncope. A tool could indicate whether a patient is at high, moderate, or low risk for serious short-term outcomes or cardiac syncope or specifically indicate whether a patient should be monitored for an extended period. Quantitative study designs and English, Dutch, German and French studies were included.

Articles were excluded when only patients with near-syncope were included. Following the criteria of Laupacis that a CDR must consist of at least three variables, studies focusing solely on one or two variables in the risk stratification of syncope patients were excluded [22]. Additionally, articles were excluded when the application of the tool, clinical decision, or follow-up time was not clearly described. Finally, case reports and narrative reviews were excluded due to limited practical usefulness and lack of clarity in evidence.

### Data extraction and synthesis

Two researchers (LB, BO) extracted the data. The researchers used a pre-set data extraction form consisting of general study characteristics and aspects specifically related to the review’s objective. The specific aspects included the tool’s name, author and year of derivation, key elements of the tool, clinical application, clinical decision, and outcomes of diagnostic or prognostic accuracy. First, the data from three articles were extracted independently by both researchers. The extracted data were compared and discussed to create a uniform method. This process was repeated until both researchers extracted ten articles’ data independently. Of the remaining studies, data were extracted by LB, where BO checked and complemented the extracted data. The data were summarised through descriptive synthesis.

Study characteristics were synthesised by study design. Subsequently, the different tools were summarised to obtain an overview of the elements and evidence per tool. Next, the elements of all tools were merged and categorised. In 2018 the European Society of Cardiology (ESC) released new guidelines for the diagnosis and management of syncope. According to these guidelines, the syncope evaluation is primarily based on three components: (1) thorough (medical) history taking, (2) physical examination, and (3) electrocardiogram [7]. Based on these findings, additional examinations may be performed. These three components of the evaluation of syncope were used as a framework for the categorisation, with the inclusion of the categories additional examinations and other variables.

### Critical appraisal

Although a quality assessment is not a mandatory element of a scoping review, we choose to add a quality assessment of included studies to give a comprehensive and more in-depth overview of the evidence on risk stratification tools for syncope patients in the chain of emergency care. A quality assessment was performed concerning the methodology of included studies, but a critical appraisal of the measurement properties was not performed. Systematic reviews were assessed using the AMSTAR-2, a 16-criteria tool [23]. Quantitative studies were assessed with a tool for different quantitative study designs developed for evaluating primary research papers in various fields with 14 criteria [24]. We deleted three criteria (criteria five, six, and seven) for experimental research because no interventions were posed within the research question. Two researchers (LB, BO) performed the quality assessment independently. A third researcher (SB) acted as a third reviewer in case of disagreement until a consensus was reached.

## Results

### Study selection

**Fig. 1:**
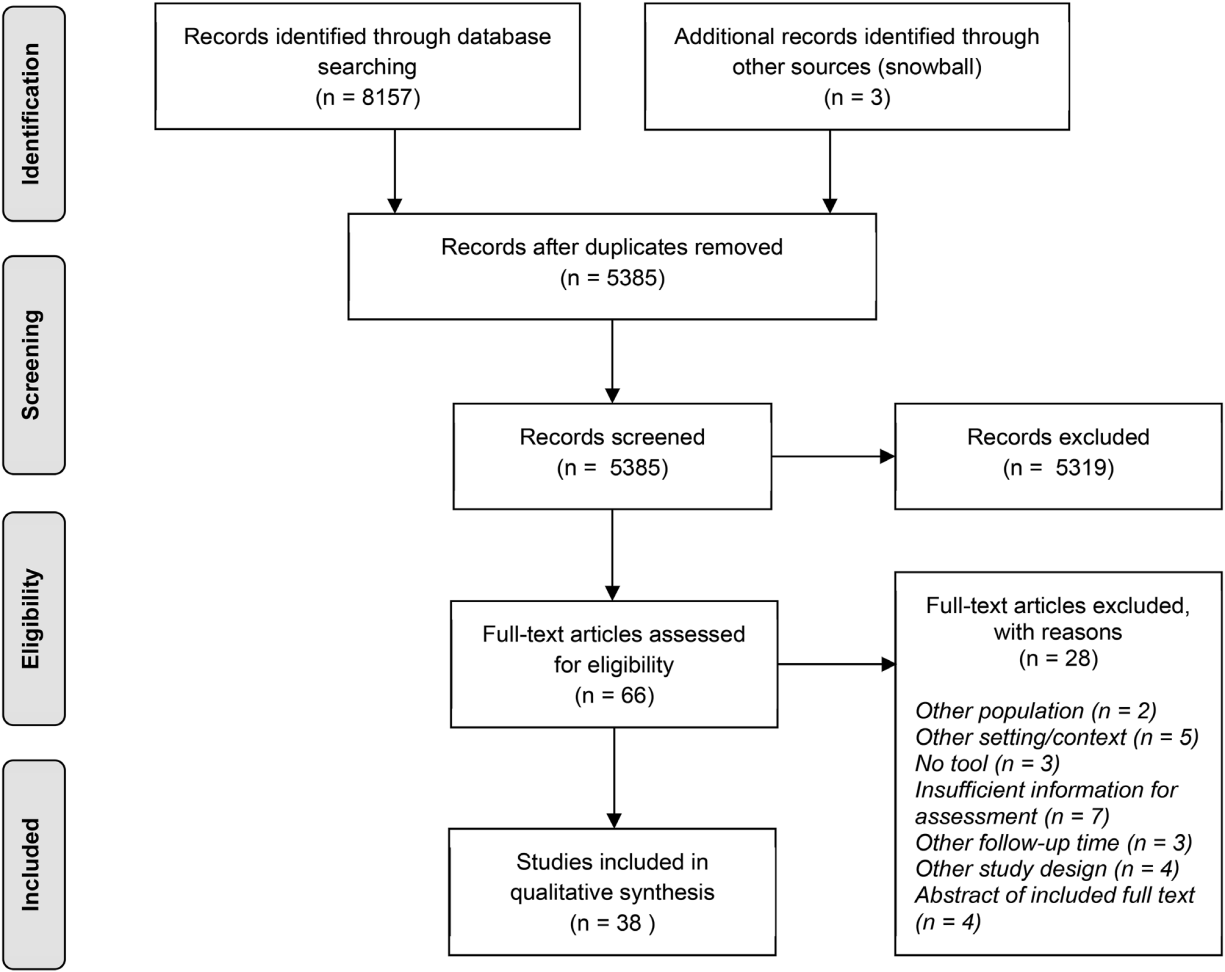
PRISMA Flow diagram

The electronic search strategy identified 5385 unique studies. After screening the title and abstract, the full text of 66 studies was assessed. Searching the included studies’ reference lists provided one study eligible for full-text assessment. In total, 38 studies were included in the review for qualitative synthesis. Figure 1 shows details of the search and selection process.

### Characteristics of included studies

The included studies concerned systematic reviews (n = 5) [14–16, 25, 26], cohort studies (n = 23) [6, 9, 27–47], electronic patient record reviews (n = 5) [48–52], and abstracts (n = 5) [53–57]. The (multicentre) studies were conducted in Germany, Italy, Spain, United Kingdom, Denmark, Switzerland, Poland, Turkey, New Zealand, Australia, Canada, United States, Colombia, Brazil, Iran, China, Israel, Saudi Arabia, and Singapore. The study population consisted of patients of various ages, and different age inclusion criteria were used, ranging from aged > 12 [41] to aged ≥ 60 [35] or age not specified [49, 53–57]. The number of patients included in the studies ranged from 62 [27] patients to 37,705 patients [49] in the individual studies. The systematic reviews included 3681 [15] to 24,234 patient(s) (visits) [16]. The follow-up period in studies varied from 48 h [6] to one month [31, 36, 40, 54–56]. The general information and results of individual studies are added in additional files 2–5.

### Quality assessment

The electronic patient record reviews, assessed with the Standard quality assessment according to Kmet et al. [24], were of good quality [49–52], except for one which was of moderate quality [48]. In all four studies of good quality, the subject characteristics were partially sufficiently described, and other elements were appropriate or sufficiently described [49–52]. The study of moderate quality did not perform well in robust and well-defined outcome measurements and did not report some estimate of variance for the main results [48]. Of the cohort studies, 21 were of good quality [6, 9, 28–33, 35–47], one of moderate quality [27], and one of poor quality [34]. The research question was partially described in eight studies [29, 31, 33, 34, 37, 40, 41, 46]. The description of subject characteristics was deemed partially sufficient in 15 studies [6, 9, 27, 28, 30, 32, 33, 35, 36, 38, 40, 42–45] and insufficient in one study [34]. The sample size was inappropriate in four studies [27, 34, 37, 40]. The studies scored well on study design, method of subject selection, and reporting of the results and conclusion. The quality of the systematic reviews, assessed using the AMSTAR-2 [23], was deemed critically low based on not reporting that the review methods were established prior to the conduct of the review and not assessing adequately for risk of bias or discussing the impact of the risk of bias on individual studies and the results [14–16, 25, 26]. We did not perform a quality assessment of the included abstracts. The complete quality assessment of the included studies can be found in additional file 6.

### Overall risk stratification tools

The included studies covered 19 tools developed between 1992 and 2020 (Table 1). Eight tools were developed before 2010 [58–65], of which one, the CHADS2 [49], was created in 2001 to stratify the risk of stroke in patients with atrial fibrillation. In 2013 Ruwald et al. used this tool to assess the risk of patients with syncope [49]. Since 2010 11 new tools have been developed [6, 29, 32, 35, 36, 42, 45, 47, 51, 52]. One tool, NEWS2-L [6], was developed in EMS patient care; the other 18 tools were developed in ED patient care. The included studies covered the derivation of a tool, validation of one tool, validation or comparison of multiple tools, and systematic reviews of one or more tools, with and without meta-analysis. Of the tools developed before 2010, multiple validation or comparison studies or systematic reviews were available of the Osservatorio Epidemiologico sulla Sincope nel Lazio (OESIL) (n = 13) [9, 14, 15, 27, 29, 32, 37, 40, 46, 53, 54, 56, 57], San Francisco Syncope Rule (SFSR) (n = 13) [9, 14, 15, 26, 27, 29, 32, 34, 37, 40, 41, 50, 54, 56, 57], Evaluation of Guidelines in Syncope Study (EGSYS) (n = 9) [15, 29, 31, 53, 54] and Boston syncope criteria (n = 7) [29, 30, 37, 48, 54, 56, 57]. The other tools were only mentioned in a systematic review, and of two tools an abstract was available [54, 56]. Only one study was available from the tools developed since 2010, except for two tools. The Risk Stratification of Syncope in the Emergency Department (ROSE) rule was described in a derivation study [36], two studies validating or comparing the ROSE rule [37, 54], and one systematic review [14]. The Canadian Syncope Risk Score (CSRS) was described in a derivation study [42] and was further validated in five studies [28, 38, 39, 44, 46].

**Table 1:** Risk stratification tools

Tool	Author (year)	Elements	Application	Clinical decision
Cardiac ischemia in syncope	Georgeson et al. (1992)	1. Ischemic abnormalities on the ECG obtained in the ED 2. Arm or shoulder pain on presentation 3. Rales on physical examination in the ED 4. Prior history of exercise-induced angina or myocardial infarction	Not described	Not described
Risk stratification in syncope	Martin et al. (1997)	1. Abnormal ECG 2. History of ventricular arrhythmia 3. History of congestive heart failure 4. Age > 45 years 5. Nonwhite race 6. No prior history of syncope	One point for each variable	Not described
Risk score to predict arrhythmias in unexplained syncope	Sarasin et al. (2003)	1. Abnormal ECG 2. Age ≥ 65 years 3. History of congestive heart failure	One point for each variable	Score 0: very low risk
OESIL	Colivicchi et al. (2003)	1. Age > 65 years 2. Cardiovascular disease in clinical history 3. Syncope without prodrome 4. Abnormal ECG	One point for each variable	Score 0–1: low risk --> outpatient evaluation and follow-up Score 2–4: high risk --> admission to the hospital
SFSR	Quinn et al. (2004)	C: Congestive heart failure H: Hematocrit < 30% E: Abnormal ECG S: Shortness of breath S: Systolic blood pressure < 90 mmHg	One point for each variable	If ≥ 1 variable is present: high risk of a serious outcome
Boston Syncope Criteria	Grossman et al. (2007)	1. Signs and symptoms of Acute Coronary Syndrome 2. Signs of conduction disease 3. Worrisome cardiac history 4. Valvular heart disease by history or physical examination 5. Family history of sudden death 6. Persistent abnormal vital signs in the ED 7. Volume depletion 8. Primary central nervous system event	One point for each variable	If any of the variables are present the patient should be admitted
EGSYS	Del Rosso et al. (2008)	1. Abnormal ECG and/or heart disease 2. Palpitations before syncope 3. Syncope during effort 4. Syncope in supine position 5. Absence of autonomic prodromes 6. Absence of predisposing and/or precipitating factors	Element 1: +3 Element 2: +4 Element 3: +3 Element 4: +2 Element 5: -1 Element 6: -1	Patients with a score ≥ 3 should be admitted
Syncope Risk Score	Sun et al. (2009)	1. Age ≥ 90 years 2. Male gender 3. History of an arrhythmia 4. Triage systolic blood pressure > 160 mmHG 5. Abnormal ECG 6. Abnormal Troponin I level 7. Complaint of near-syncope	Element 1–6: +1 Element 7: -1	Score − 1 − 0: low risk Score 1–2: intermediate risk Score 3–6: high risk
ROSE	Reed et al. (2010)	B: BNP level > 300pv/ml or Bradycardia < 50/min (in ED or prehospital) R: Rectal examination showing fecal occult blood A: Anemia, HB < 90 g/L C: Chest pain associated with syncope E: ECG showing Q wave (not in lead III) S: Saturation < 94% - room air	One point for each variable	If ≥ 1 variable is present: high risk of a serious outcome Consider admission if ≥ 1 variable is present
Anatolian Syncope Rule	Kayayurt et al. (2012)	D: Dyspnoea O: Ortostatism P: Precipitating cause for syncope A: Age > 58 years C: Congestive heart failure history E: ECG abnormality	Element D - C: +1 Element E: +2	A score > 1: high risk syncope A score > 2: high risk mortality
Ottawa Electrocardiographic Criteria	Thiruganasambandamoorthy et al. (2012)	Based on ECG of the patient 1. Blocks: a. Second-degree Mobitz type 2 or third-degree AV block b. Bundle branch block + first-degree AV block c. Right bundle branch + left anterior or posterior fascicular block 2. New ischemic changes 3. Nonsinus rhythm 4. Left axis deviation 5. ED cardiac monitor abnormalities	One point for each variable	If ≥ 1 variable is present: high risk of a serious outcome
CHADS2 score	Ruwald et al. (2013)	C: Chronic heart failure H: Hypertension A: Age ≥ 75 years D: Diabetes S: Prior transient ischemic attack or stroke	Element C - D: +1 Element S: +2	Not described
Syncope Risk Scale	Thiruganasambandamoorthy et al. (2014)	1. Age ≥ 75 years 2. Shortness of breath 3. Lowest ED systolic BP < 80 mmHG 4. The presence of the Ottawa Electrocariographic Criteria 5. BUN > 15 mmol/L	Element 1: +1 Element 2: +2 Element 3: +2 Element 4: +2 Element 5: +3	Score 0: low risk Score 1: moderate risk Score ≥ 2: high risk
CSRS	Thiruganasambandamoorthy et al. (2016)	1. Predisposition to vasovagal syncope 2. Heart disease 3. Any systolic pressure in the ED < 90 or > 180 mmHG 4. Troponin level > 99th percentile for the normal population 5. Abnormal QRS axis (<-30° of > 100°) 6. QRS duration > 130 ms 7. QTc interval > 480 ms 8. ED diagnosis of cardiac syncope 9. ED diagnosis of vasovagal syncope	Element 1: -1 Element 2: +1 Element 3: +2 Element 4: +2 Element 5: +1 Element 6: +1 Element 7: +2 Element 8: +2 Element 9: -2	Score − 3 - -2: very low risk Score − 1 − 0: low risk Score 1–3: medium risk Score 4–5: high risk Score 6–11: very high risk
IC-FUC score	Gomes et al. (2016)	1. Previous history of syncope 2. Known heart disease 3. Abnormal ECG	Element 1: +2 Element 2: +4 Element 3: +3	Not described
Canadian Syncope Arrhythmia Risk Score	Thiruganasambandamoorthy et al. (2017)	1. Vasovagal predisposition 2. History of heart disease 3. Any ED systolic BP < 90 or > 180mmHG 4. Troponin elevated (> 99%ile normal population) 5. QRS duration > 130 ms 6. Corrected QT interval > 480 ms 7. ED diagnosis of vasovagal syncope 8. ED diagnosis of cardiac syncope	Element 1: -1 Element 2: +1 Element 3: +1 Element 4: +1 Element 5: +2 Element 6: +1 Element 7: -1 Element 8: +2	Score − 2 − 0: very low risk Score 1: low risk Score 2–3: medium risk Score 4–5: high risk Score 6–8: very high risk
NEWS2-L	Martín-Rodriquez et al. (2020)	1. NEWS2 - Heart rate (0–3 points) - Breathing rate (0–3 points) - Temperature (0–3 points) - Systolic blood pressure (0–3 points) - Oxygen saturation (0–3 points) - Air oxygen (0–2 points) - AVPU (0–3 points) 2. pLA	Element 1: numerical value of all determinants together Element 2: numerical value of the test	A score ≥ 6.9: high risk syncope
FAINT score	Probst et al. (2020)	F: History of heart Failure A: History of cardiac Arrhythmia I: Abnormal Initial ECG N: Elevated NT-pro-BNP level T: Elevated hs-cTnT level	Element N: +2 Other elements: +1	Score > 0: high risk syncope
ALERT-CS	Zimmerman et al. (2021)	1. Rhythm 2. Heart rate 3. Corrected QT-interval 4. ST-segment depression 5. Atrioventricular-block 6. Bundle-branch-block 7. Ventricular extrasystole/non-sustained ventricular tachycardia	Computational calculation of probability of cardiac cause of syncope	Rule-in high risk: 37.5% Rule-out: <5.5%

Abbreviations: *CSRS* Canadian Syncope Risk Score, *ECG* electrocardiogram, *ED* emergency department, *EGSYS* Evaluation of Guidelines in Syncope Study, *NEWS* National Early Warning Signs, *OESIL* Osservatorio Epidemiologico sulla Sincope nel Lazio, *pLA* point-of-care lactate measurement, *ROSE* Risk Stratification of Syncope in the Emergency Department, *SFSR* San Francisco Syncope Rule

In addition to validating or comparing tools, three studies evaluated the value of adding a laboratory result to an existing tool. This evaluation involves adding the value of S100B to the OESIL and SFSR [27], the value of B-type natriuretic peptide (BNP), N-terminal proBNP (NT-proBNP), and high-sensitive cardiac troponin (hs-cTn) T and I to the EGSYS, ROSE, OESIL, SFSR, CSRS [33] and the value of NT-proBNP to the CSRS [43]. One study evaluated the value of adding echocardiography to patients stratified as moderate-high risk by the OESIL [55].

### Outcome measures of the studies

The outcome measures used in the included studies are divided into prognostic endpoints and diagnostic outcomes. Prognostic endpoints were aimed at serious short-term outcomes within the follow-up time and included diverse cardiac events, (major) therapeutic procedures, pulmonary embolus, severe infection/sepsis, cerebrovascular accidents, intracranial bleeding, haemorrhage, intensive care unit admission, and readmission and death. The diagnostic outcome focused on the diagnosis of non-cardiogenic or cardiogenic syncope.

### Application of risk stratification tools

The different risk stratification tools with their elements, application in practice, and subsequent clinical decisions are presented in Table 1. One element consisted of one to seven variables. The tools contained three to 25 variables, divided into two to nine elements. There were two main approaches for the application and the clinical decision regarding the different tools. A score was awarded to each element in the first approach (n = 9) [6, 32, 35, 42, 45, 52, 61, 64, 65]. These scores ranged from minus two to four, except for the point-of-care lactate test (pLA) of NEWS2-L. Of the pLA, the specific value given by the test was used [6]. The scores of all elements were added up to provide an end score. Based on this end score, a patient was classified as having a high, medium, or low risk of a serious short-term outcome or an origin of cardiac syncope. In general, the higher the score, the greater the risk. In the second approach (n = 4) [36, 51, 62, 63], a patient was classified as having a high risk of a serious short-term outcome when one or more elements were present. One tool, the ALERT-CS, worked with a calculator. The electrocardiogram (ECG) criteria were entered into a computer program. After which, the computer program shows the probability of (1) serious short-term outcomes and (2) a cardiac cause of syncope [47]. Of five tools, no clear description of the application or clinical decision based on the elements of the tool was available [29, 49, 58–60].

### Elements of tools

The studies described a total of 104 elements, which represent multiple variables. The variables were categorised according to the components of syncope evaluation [7], and analysis revealed several subcategories. The distribution of categories per original tool is displayed in Table 2.

**Table 2:** Distribution of categories per tool

	History taking			Physical examination			ECG	Add. examinations	Other
	*Medical history*	*History of event*	*Demographic data*	*Cardiac*	*Pulmonary*	*Vital signs*			
Cardiac ischemia in syncope (1992)	X			X	X		X		
Risk stratification in syncope (1997)	X		X				X		
Risk score to predict arrhythmias in unexplained syncope (2003)	X		X				X		
OESIL (2003)	X	X	X				X		
SFSR (2004)	X				X	X	X	X	
Boston Syncope Criteria (2007)	X	X		X	X	X	X	X	X
EGSYS (2008)	X	X					X		
Syncope Risk Score (2009)	X	X	X			X	X	X	
ROSE (2010)		X				X	X	X	X
Anatolian Syncope Rule (2012)	X	X	X	X	X		X		
Ottawa Electrocardiographic Criteria (2012)							X		
CHADS2 score (2013)	X		X						
Syncope Risk Scale (2014)			X		X	X	X	X	
CSRS (2016)	X					X	X	X	X
IC-FUC score (2016)	X						X		
Canadian Syncope Arrhythmia Risk Score (2017)	X					X	X	X	X
NEWS2-L (2020						X		X	
FAINT score (2020)	X						X	X	
ALERT-CS (2021)							X		

Abbreviations: *CSRS* Canadian Syncope Risk Score, *EGSYS* Evaluation of Guidelines in Syncope Study, *OESIL* Osservatorio Epidemiologico sulla Sincope nel Lazio, *ROSE* Risk Stratification of Syncope in the Emergency Department, *SFSR* San Francisco Syncope Rule

- History taking.
  - Medical history – a history of heart disease(s), such as congestive heart failure, valvular heart disease, arrhythmia or use of anti-dysrhythmic medication, was often included in the tools (n = 14) [29, 32, 35, 42, 45, 49, 58–65]. History of syncope was another component of the medical history and was present in five tools [29, 42, 45, 59, 63]. A history of diabetes was mentioned in one tool [49].
  - History of the event – this subcategory included symptoms related to the syncope incident, such as the patient’s position during syncope, the presence of prodromes, and chest pain associated with syncope. A total of six tools contained a variable concerning the history of the event [32, 36, 61, 63–65], where five out of six elements of the EGSYS were based on the history of the event [64].
  - Demographic data – seven tools contained demographic data of race, gender, or age [32, 49, 52, 59–61, 65]. Age as demographic data was used in all seven tools. The cut-off value ranged from > 45 to ≥ 90 years.
- Physical examination.
  - Cardiac variables – signs and symptoms of cardiac disease related to the event, such as arm or shoulder pain, signs of volume depletion, and orthostatism, were included in three tools [32, 58, 63].
  - Pulmonary variable – five tools contained a pulmonary variable directly related to the syncope, such as rales or dyspnea [32, 52, 58, 62, 63].
  - Vital signs – general and specific values of vital signs were included in eight tools [6, 36, 42, 45, 52, 62, 63, 65], of which systolic blood pressure was most present.
- Electrocardiogram (ECG) – a variable related to the ECG was present in 17 tools. Nine tools included the variable “abnormal ECG” without further specification [29, 32, 35, 59–62, 64, 65]. Eight tools included one or more specific ECG abnormalities in their tool [36, 42, 45, 47, 51, 52, 58, 63], of which the ALERT-CS [47] was based entirely on specific ECG abnormalities.
- Additional examinations.
  - Laboratory results – specific laboratory results, such as hematocrit, NT-proBNP, or troponin, were included in nine original tools [6, 35, 36, 42, 45, 52, 62, 63, 65]. Specific values for laboratory results are given or specified as being ‘elevated’. Laboratory results are also added to original tools in three studies [27, 33, 43].
  - Additional tests – additional tests were not included in the original tools. One study evaluated the value of adding echocardiography to the OESIL [55].
- Other variables – four tools described other variables: a primary central nervous system event (i.e., subarachnoid haemorrhage, stroke), ED diagnosis of cardiac or vasovagal syncope, and signs of gastrointestinal bleeding [36, 42, 45, 63].

### Measurement properties of the tools

The measurement properties used were mainly focused on validity, with particular use of the properties sensitivity and specificity. These measurement properties were used in > 80% of studies. The positive and negative predictive values were used in half of the studies. In about one-third of the studies, the positive likelihood ratio (LRP), negative likelihood ratio (LRN), and the area under the curve (AUC) were calculated. Usually, more than one measurement property was presented, except in the AUC. The AUC was used as a single measurement property and in combination with other measurement properties.

## Discussion

We identified 38 studies with 19 risk stratification tools for patients with syncope in EMS and ED patient care, including four studies evaluating the value of adding an extra variable to an already existing tool. The risk stratification tools are primarily developed within the ED, with only one tool being derived in EMS patient care. A total of 104 elements were discovered, of which elements indicating a possible cardiac problem can be identified as key elements. In addition, we found two main approaches in the application and consequent clinical decision of the tools. In the first approach, a score was awarded to each element, and the scores of all elements were added up to provide an end score. Based on this end score, a patient was classified as having a high, medium, or low risk of a serious short-term outcome. In the second approach, a patient was classified as having a high risk of serious short-term outcomes when one or more elements were present.

The number of risk stratification tools identified in this scoping review substantially exceeds those from earlier reviews [14–16]. This increase in number can be explained by the purpose of a scoping review, in which it is possible to generate a broad overview and include more studies than previous systematic reviews. The wide variety of existing tools could implicate a wide variation in risk stratification and clinical decision-making in syncope patient care. This leads to a potential risk to patient safety. In addition, this broad overview is reflected in the associated studies of the identified tools. We found multiple studies for only six out of 19 tools [36, 42, 61–64]. For the other tools, only one study was described. The fact that 13 tools have been developed that are not further investigated, validated, or integrated into clinical practice is intriguing and disturbing. The lack of external validation, combined with the complexity of use, various use of outcome measures and paucity of data showing improved clinical outcomes compared to clinical judgement, could be reasons tools were not widely accepted in clinical practice [7, 15, 66, 67]. Therefore, the demand for a risk stratification tool remained, which could have led to the continued development of new tools.

Syncope does not seem unique as a disorder with multiple risk stratification tools. In acute care, several risk stratification tools often exist for the same disorder or symptom, such as sepsis, general surgery, chest pain, or frailty in the elderly [68–71]. A systematic review identifying evidence on the feasibility of risk stratification tools assessing frailty in the elderly in the ED showed that even though tools seem feasible, adequate implementation in clinical practice remains challenging. They indicate that additional work is required to understand how professionals will likely use tools and when to ensure they are acceptable in emergency care [69]. In addition, to aid implementation in clinical practice, it could be helpful to consider how professionals operate from a behavioural and cultural perspective. One can think of Kahnemann’s theory of intuition and reasoning [72], the theory of Shein regarding organisational culture and leadership [73], or implementation strategies according to Grol and Wensing [74]. However, further elaboration on implementation is beyond the scope of this scoping review. Nevertheless, successful implementation and dissemination is essential and requires tools optimally fitted to the context of (pre)hospital emergency care. Otherwise, if the need for risk stratification support is not adequately met, the development and derivation of new tools may be stimulated and will continue. Allowing variation in patient care to persist with potential risks.

The key element in the risk stratification of syncope patients seems to include elements related to potential cardiac problems. Only two tools did not include an element directly related to possible problems of cardiac origin [6, 49]. The electrocardiogram was most present in the tools (n = 17), followed by a medical history of heart disease(s) (n = 14). The emphasis on cardiac problems is consistent with the European and American guidelines for the diagnosis and management of syncope, where the risk of a cardiovascular event plays a significant role in the evaluation, especially in the early risk stratification regarding the management of syncope in the acute setting [7, 75].

There are significantly more risk stratification tools developed in ED patient care compared to EMS, and these tools are often not directly transferrable to the EMS due to the requirements of additional examinations, such as laboratory tests. Although point-of-care measurements exist in EMS, this is often limited to research studies [76, 77]. The lack of possibilities for additional examinations in EMS patient care makes risk stratification and decision-making in prehospital care even more complex. In addition, other key elements could be relevant in the EMS context because, upon arrival of the EMS professional, the incident has recently happened, compared to the longer period that has passed upon the patient’s presentation at the ED. Investigating and understanding key elements relevant to the EMS is essential to develop a tailored EMS protocol or tool to reduce overtriage and prevent undertriage in patients with syncope. A tailored EMS protocol or tool seems urgent as approximately 40% of syncope patients transported to the ED have shown to be at low risk and appear not to require ED assessment [12].

This scoping review has generated an overview of 19 risk stratification tools, most of which have not been further validated. Therefore, further research should aim to reach a consensus on which risk stratification tools are estimated to have the best impact and support risk stratification and decision-making in syncope patients in (pre)hospital emergency care. In future studies, the specific context and possible differences between EMS and ED patient care should be considered beforehand to develop and generate tailored or modified risk stratification tools for the EMS and ED setting. Moreover, the care for syncope patients should be approached from a multidisciplinary medical perspective to ensure that risk stratification and decision-making in the chain of emergency care are aligned. In addition, the (modified) risk stratification tools should be critically appraised regarding the relevant measurement properties following the COSMIN. Appropriate validation based on comparison with clinical judgement is essential here. If a risk stratification tool is deemed applicable and relevant, it should be integrated and implemented into guidelines regarding the emergency care management of syncope patients.

The limitations of this review are partly inherently linked to the design of a scoping review. To generate a general overview of the methodology of the studies, we performed a generic quality assessment. However, we did not perform a quality assessment of the measurement properties or a quality assessment focusing on the development of tools. We cannot make assumptions about the tools’ rigour, validity, or reliability by not using a specific quality assessment for tools. However, this was not part of our aim. Another limitation is related to the search strategy. We included a broad range of evidence sources, but we did not search the grey literature, contact authors of primary sources, or include unpublished data. Otherwise, possibly even more tools would have been found. However, this could have led to even less scientifically designed tools.

## Conclusion

A total of 19 risk stratification tools developed for syncope patients were identified, of which most were not validated. The risk stratification tools were primarily established in ED patient care, with only one tool derived in EMS patient care. Key elements in the risk stratification were related to a potential cardiac problem as the cause of the syncope. The wide variety of, mostly not validated, tools could lead to a risk to patient safety. To enhance patient safety and to support professionals in risk stratification, consensus should be reached regarding the risk stratification tools deemed most relevant and applicable in the chain of emergency care. Subsequently, appropriate validation and assessment of the measurement properties of these tool(s) should be performed. In addition, the differences in the context and treatment possibilities in (pre)hospital EMS and ED patient care should be considered in assessing and developing tools. Given the gap between risk stratification tools for ED and EMS patient care, the initial focus should be on a protocol or tool for EMS patient care to reduce overtriage while preventing undertriage. Lastly, there should be an emphasis on a sound implementation strategy.

### Electronic supplementary material

Below is the link to the electronic supplementary material.

Additional file 1: Search strategies

Additional file 2: Characteristics of included systematic reviews

Additional file 3: Characteristics of included cohort studies

Additional file 4: Characteristics of included electronic patient record reviews

Additional file 5: Characteristics of included abstracts

Additional file 6: Quality assessment of included studies

## Data Availability

All data generated or analysed during this study are included in this published article (and it’s supplementary files).

## Abbreviations

AUC: area under the curve
BNP: B-type natriuretic peptide
CDR: Clinical Decision Rule
CINAHL: Cumulative Index to Nursing and Allied Health Literature
CSRS: Canadian Syncope Risk Score
ECG: Electrocardiogram
ED: Emergency Department
EGSYS: Evaluation of Guidelines in Syncope Study
EMS: Emergency Medical Services
ESC: European Society of Cardiology
hs-cTn: high-sensitive cardiac troponin
LRP: positive likelihood ratio
LRN: negative likelihood ratio
NEWS: National Early Warning Signs
NT-proBNP: N-terminal proBNP
pLA: point-of-care lactate measurement
PRISMA-ScR: Preferred Items for Systematic Reviews and Meta-analysis Extension for Scoping Review
OESIL: Osservatorio Epidemiologico sulla Sincope nel Lazio
ROSE: Risk Stratification of Syncope in the Emergency Department
SFSR: San Francisco Syncope Rule
T-LOC: Transient Loss of Consciousness
