# Supplementary material for: Risk stratification tools for patients with syncope in emergency medical services and emergency departments: a scoping review
Source: Scand J Trauma Resusc Emerg Med. 2023 Sep 18;31:48. doi: 10.1186/s13049-023-01102-z (PMC10508018; doi:10.1186/s13049-023-01102-z)
Supplement: Supplementary file 1 — Additional file 1: Search strategies [file 13049_2023_1102_MOESM1_ESM.pdf]

Additional file 1

Search strategy

Medline (EBSCO)

S1 MH "Syncope+" OR TI (syncop* OR presyncope* OR (Drop N1 Attack*) OR fainting) OR SU (syncop* OR presyncope* OR (Drop N1 Attack*) OR fainting)

S2 MH "Triage" OR TI triag*

S3 S1 AND S2

S4 MH "Emergency Medicine+" OR MH "Emergency Medical Services" OR MH "Transportation of Patients+" OR MH "Emergency Service, Hospital+" OR MH "Advanced Trauma Life Support Care" OR MH "Emergency Medical Technicians" OR TI ((Emergenc* N1 (Service* OR department* OR medicine* OR care OR centre* OR center*)) OR (trauma N1 (center* OR centre*)) OR (Emergency N1 (Unit* OR ward* OR room*)) OR EMS OR ((prehospital* or “pre hospital”) N2 care) OR EMT OR (emergency N2 medical N2 technician*) OR paramedic* OR ((emergency OR Rescue OR recovery OR EMS) N2 (worker* OR personnel OR provider* OR staff OR practitioner* OR nurs*))) OR AB ((Emergenc* N1 (Service* OR department* OR medicine* OR care OR centre* OR center*)) OR (trauma N1 (center* OR centre*)) OR (Emergency N1 (Unit* OR ward* OR room*)) OR EMS OR ((prehospital* or “pre hospital”) N2 care) OR EMT OR (emergency N2 medical N2 technician*) OR paramedic* OR ((emergency OR Rescue OR recovery OR EMS) N2 (worker* OR personnel OR provider* OR staff OR practitioner* OR nurs*))) OR SU ((Emergenc* N1 (Service* OR department* OR medicine* OR care OR centre* OR center*)) OR (trauma N1 (center* OR centre*)) OR (Emergency N1 (Unit* OR ward* OR room*)) OR EMS OR ((prehospital* or “pre hospital”) N2 care) OR EMT OR (emergency N2 medical N2 technician*) OR paramedic* OR ((emergency OR Rescue OR recovery OR EMS) N2 (worker* OR personnel OR provider* OR staff OR practitioner* OR nurs*)))

S5 MH "Syncope+/DI" OR TI ((drop N1 attack) OR syncope OR presyncope) N5 (diagnosis OR asses* OR screen* OR predict* OR score* OR checklist* OR risk* OR rule* OR measur*) OR AB ((drop N1 attack) OR syncope OR presyncope) N5 (diagnosis OR asses* OR screen* OR predict* OR score* OR checklist* OR risk* OR rule* OR measur*) OR SU ((drop N1 attack) OR syncope OR presyncope) N5 (diagnosis OR asses* OR screen* OR predict* OR score* OR checklist* OR risk* OR rule* OR measur*)

S6 S4 AND S5

S7 TI ((syncope N3 (score* OR rule* OR measur*)) OR “Osservatorio Epidemiologico sulla Sincope nel Lazio” OR (syncope N3 (rose OR oesil OR SFSR OR CSRS))) OR AB ((syncope N3 (score* OR rule* OR measur*)) OR “Osservatorio Epidemiologico sulla Sincope nel Lazio” OR (syncope N3 (rose OR oesil OR SFSR OR CSRS))) OR SU ((syncope N3 (score* OR rule* OR measur*)) OR “Osservatorio Epidemiologico sulla Sincope nel Lazio” OR (syncope N3 (rose OR oesil OR SFSR OR CSRS)))

S8 MH "Clinical Decision-Making" OR MH "Decision Support Techniques+" OR MH "Decision Making+" OR MH "Risk Assessment+" OR TI ((Risk N1 (Stratificati* OR assess*)) OR (Decision N1 Support N1 Technique*) OR (“Risk-Stratification”) OR (Clinical N1 Decision N1 (Rule* OR making)) OR (Clinical N1 Prediction N1 Guid*) OR Prognosis OR (Serious N1 outcome*) OR (Serious N1 adverse N1 event*) OR (Risk N1 scale*) OR Algorithm*) OR AB ((Risk N1 (Stratificati* OR assess*)) OR (Decision N1 Support N1 Technique*) OR (“Risk-Stratification”) OR (Clinical N1 Decision N1 (Rule* OR making)) OR (Clinical N1 Prediction N1 Guid*) OR Prognosis OR (Serious N1 outcome*) OR (Serious N1 adverse N1 event*) OR (Risk N1 scale*) OR Algorithm*) OR SU ((Risk N1 (Stratificati* OR assess*)) OR (Decision N1 Support N1 Technique*) OR (“Risk-Stratification”) OR (Clinical N1 Decision N1 (Rule* OR making)) OR (Clinical N1 Prediction N1 Guid*) OR Prognosis OR (Serious N1 outcome*) OR (Serious N1 adverse N1 event*) OR (Risk N1 scale*) OR Algorithm*)

S9	S1 AND S8

S10	S3 OR S6 OR S7 OR S9

<u>CINAHL+ with full text (EBSCO)</u>

S1	MH "Syncope+" OR TI (syncop* OR presyncope* OR (Drop N1 Attack*) OR fainting) OR SU (syncop* OR presyncope* OR (Drop N1 Attack*) OR fainting)

S2	MH "Triage" OR TI triag*

S3	S1 AND S2

S4	MH "Emergency Medicine" OR MH "Emergency Medical Services" OR MH "Emergency Service" OR MH "Emergency Services, Psychiatric" OR MH "Transportation of Patients+" OR MH "Trauma Centers" OR MH "Advanced Trauma Life Support Care" OR MH "Emergency Medical Technicians" OR TI ((Emergenc* N1 (Service* OR department* OR medicine* OR care OR centre* OR center*)) OR (trauma N1 (center* OR centre*)) OR (Emergency N1 (Unit* OR ward* OR room*)) OR EMS OR ((prehospital* or “pre hospital”) N2 care) OR EMT OR (emergency N2 medical N2 technician*) OR paramedic* OR ((emergency OR Rescue OR recovery OR EMS) N2 (worker* OR personnel OR provider* OR staff OR practitioner* OR nurs*))) OR AB ((Emergenc* N1 (Service* OR department* OR medicine* OR care OR centre* OR center*)) OR (trauma N1 (center* OR centre*)) OR (Emergency N1 (Unit* OR ward* OR room*)) OR EMS OR ((prehospital* or “pre hospital”) N2 care) OR EMT OR (emergency N2 medical N2 technician*) OR paramedic* OR ((emergency OR Rescue OR recovery OR EMS) N2 (worker* OR personnel OR provider* OR staff OR practitioner* OR nurs*))) OR SU ((Emergenc* N1 (Service* OR department* OR medicine* OR care OR centre* OR center*)) OR (trauma N1 (center* OR centre*)) OR (Emergency N1 (Unit* OR ward* OR room*)) OR EMS OR ((prehospital* or “pre hospital”) N2 care) OR EMT OR (emergency N2 medical N2 technician*) OR paramedic* OR ((emergency OR Rescue OR recovery OR EMS) N2 (worker* OR personnel OR provider* OR staff OR practitioner* OR nurs*)))

S5	MH "Syncope+/DI" OR TI ((drop N1 attack) OR syncope OR presyncope) N5 (diagnosis OR asses* OR screen* OR predict* OR score* OR checklist* OR risk* OR rule* OR measur*) OR AB ((drop N1 attack) OR syncope OR presyncope) N5 (diagnosis OR asses* OR screen* OR predict* OR score* OR checklist* OR risk* OR rule* OR measur*) OR SU ((drop N1 attack) OR syncope OR presyncope) N5 (diagnosis OR asses* OR screen* OR predict* OR score* OR checklist* OR risk* OR rule* OR measur*)

S6	S4 AND S5

S7	TI ((syncope N3 (score* OR rule* OR measur*)) OR “Osservatorio Epidemiologico sulla Sincope nel Lazio” OR (syncope N3 (rose OR oesil OR SFSR OR CSRS))) OR AB ((syncope N3 (score* OR rule* OR measur*)) OR “Osservatorio Epidemiologico sulla Sincope nel Lazio” OR (syncope N3 (rose OR oesil OR SFSR OR CSRS))) OR SU ((syncope N3 (score* OR rule* OR measur*)) OR “Osservatorio Epidemiologico sulla Sincope nel Lazio” OR (syncope N3 (rose OR oesil OR SFSR OR CSRS)))

S8	MH "Decision Making, Clinical" OR MH "Decision Support Systems, Clinical" OR MH "Decision Support Techniques+" OR MH "Decision Making" OR MH "Decision Making, Shared" OR MH "Risk Assessment" OR TI ((Risk N1 (Stratificati* OR assess*)) OR (Decision N1 Support N1 Technique*) OR (“Risk-Stratification”) OR (Clinical N1 Decision N1 (Rule* OR making)) OR (Clinical N1 Prediction N1 Guid*) OR Prognosis OR (Serious N1 outcome*) OR (Serious N1 adverse N1 event*) OR (Risk N1 scale*) OR Algorithm*) OR AB ((Risk N1 (Stratificati* OR assess*)) OR (Decision N1 Support N1 Technique*) OR (“Risk-Stratification”) OR (Clinical N1 Decision N1 (Rule* OR making)) OR (Clinical N1 Prediction N1 Guid*) OR Prognosis OR (Serious N1 outcome*) OR (Serious N1 adverse N1 event*) OR (Risk N1 scale*) OR Algorithm*) OR SU ((Risk N1 (Stratificati* OR assess*)) OR (Decision N1 Support

N1 Technique*) OR ("Risk-Stratification") OR (Clinical N1 Decision N1 (Rule* OR making)) OR (Clinical N1 Prediction N1 Guid*) OR Prognosis OR (Serious N1 outcome*) OR (Serious N1 adverse N1 event*) OR (Risk N1 scale*) OR Algorithm*)

S9 S1 AND S8

S10 S3 OR S6 OR S7 OR S9

<u>Embase.com (OVID)</u>

1 heat syncope/ or presyncope/ OR (syncop* OR presyncope* OR (Drop ADJ1 Attack*) OR fainting).ti,kw.

2 triag*.ti.

3 1 AND 2

4 exp emergency medicine/ OR exp emergency health service/ OR exp patient transport/ OR exp emergency care/ OR rescue personnel/ OR ((Emergenc* ADJ1 (Service* OR department* OR medicine* OR care OR centre* OR center*)) OR (trauma ADJ1 (center* OR centre*)) OR (Emergency ADJ1 (Unit* OR ward* OR room*)) OR EMS OR ((prehospital* or "pre hospital") ADJ2 care) OR EMT OR (emergency ADJ2 medical ADJ2 technician*) OR paramedic* OR ((emergency OR Rescue OR recovery OR EMS) ADJ2 (worker* OR personnel OR provider* OR staff OR practitioner* OR nurs*))).ti,ab,kw.

5 exp faintness/di OR ((drop ADJ1 attack) OR syncope OR presyncope) ADJ5 (diagnosis OR asses* OR screen* OR predict* OR score* OR checklist* OR risk* OR rule* OR measur*).ti,ab,kw.

6 4 AND 5

7 ((syncope ADJ3 (score* OR rule* OR measur*)) OR "Osservatorio Epidemiologico sulla Sincope nel Lazio" OR (syncope ADJ3 (rose OR oesil OR SFSR OR CSRS))).ti,ab,kw.

8 decision making/ or exp clinical decision making/ or medical decision making/ or shared decision making/ OR clinical decision support system/ OR exp decision support system/ OR risk assessment/ OR ((Risk ADJ1 (Stratificati* OR assess*)) OR (Decision ADJ1 Support ADJ1 Technique*) OR ("Risk-Stratification") OR (Clinical N1 Decision ADJ1 (Rule* OR making)) OR (Clinical ADJ1 Prediction ADJ1 Guid*) OR Prognosis OR (Serious ADJ1 outcome*) OR (Serious ADJ1 adverse ADJ1 event*) OR (Risk ADJ1 scale*) OR Algorithm*).ti,ab,kw.

9 1 AND 8

10 3 OR 6 OR 7 OR 9

11 limit 10 to conference abstract status

12 10 NOT 11

<u>Cochrane central</u>

#1 syncop* OR presyncope* OR (Drop NEAR/1 Attack*) OR fainting):ti,kw

#2 triag*:ti

#3 #1 AND #2

#4 ((Emergenc* NEAR/1 (Service* OR department* OR medicine* OR care OR centre* OR center*)) OR (trauma NEAR/1 (center* OR centre*)) OR (Emergency NEAR/1 (Unit* OR ward*

OR room*)) OR EMS OR ((prehospital* or “pre hospital”) NEAR/2 care) OR EMT OR (emergency NEAR/2 medical NEAR/2 technician*) OR paramedic* OR ((emergency OR Rescue OR recovery OR EMS) NEAR/2 (worker* OR personnel OR provider* OR staff OR practitioner* OR nurs*))):ti,ab,kw

#5 ((drop NEAR/1 attack) OR syncope OR presyncope) NEAR/5 (diagnosis OR asses* OR screen* OR predict* OR score* OR checklist* OR risk* OR rule* OR measur*):ti,ab,kw

#6 #4 AND #5

#7 TS=((syncope NEAR/3 (score* OR rule* OR measur*)) OR “Osservatorio Epidemiologico sulla Sincope nel Lazio” OR (syncope ADJ3 (rose OR oesil OR SFSR OR CSRS))):ti,ab,kw

#8 ((Risk NEAR/1 (Stratificati* OR assess*)) OR (Decision NEAR/1 Support NEAR/1 Technique*) OR (“Risk-Stratification”) OR (Clinical N1 Decision NEAR/1 (Rule* OR making)) OR (Clinical NEAR/1 Prediction NEAR/1 Guid*) OR Prognosis OR (Serious NEAR/1 outcome*) OR (Serious NEAR/1 adverse NEAR/1 event*) OR (Risk NEAR/1 scale*) OR Algorithm*):ti,ab,kw

#9 #1 AND #8

#10 #3 OR #6 OR #7 OR #9

<u>Web of Science Core Collection</u>

#1 TS=(syncop* OR presyncope* OR (Drop NEAR/1 Attack*) OR fainting)

#2 TI=triag*

#3 #1 AND #2

#4 TS=((Emergenc* NEAR/1 (Service* OR department* OR medicine* OR care OR centre* OR center*)) OR (trauma NEAR/1 (center* OR centre*)) OR (Emergency NEAR/1 (Unit* OR ward* OR room*)) OR EMS OR ((prehospital* or “pre hospital”) NEAR/2 care) OR EMT OR (emergency NEAR/2 medical NEAR/2 technician*) OR paramedic* OR ((emergency OR Rescue OR recovery OR EMS) NEAR/2 (worker* OR personnel OR provider* OR staff OR practitioner* OR nurs*)))

#5 TS=((drop NEAR/1 attack OR syncope OR presyncope) NEAR/5 (diagnosis OR asses* OR screen* OR predict* OR score* OR checklist* OR risk* OR rule* OR measur*))

#6 #4 AND #5

#7 TS=((syncope NEAR/3 (score* OR rule* OR measur*)) OR “Osservatorio Epidemiologico sulla Sincope nel Lazio” OR (syncope ADJ3 (rose OR oesil OR SFSR OR CSRS)))

#8 TS=((Risk NEAR/1 (Stratificati* OR assess*)) OR (Decision NEAR/1 Support NEAR/1 Technique*) OR (“Risk-Stratification”) OR (Clinical N1 Decision NEAR/1 (Rule* OR making)) OR (Clinical NEAR/1 Prediction NEAR/1 Guid*) OR Prognosis OR (Serious NEAR/1 outcome*) OR (Serious NEAR/1 adverse NEAR/1 event*) OR (Risk NEAR/1 scale*) OR Algorithm*)

#9 #1 AND #8

#10 #3 OR #6 OR #7 OR #9
